# Commentary: United States Dietary Trends Since 1800: Lack of Association Between Saturated Fatty Acid Consumption and Non-communicable Diseases

**DOI:** 10.3389/fnut.2022.891792

**Published:** 2022-04-28

**Authors:** Wendy Walrabenstein, Catharina S. de Jonge, Anna M. Kretova, Marieke van de Put, Carlijn A. Wagenaar, Franktien Turkstra, Hana Kahleova, Simon J. Hill, Dirkjan van Schaardenburg

**Affiliations:** ^1^Amsterdam Rheumatology & Immunology Center, Reade, Amsterdam, Netherlands; ^2^Amsterdam Rheumatology & Immunology Center, Amsterdam UMC, University of Amsterdam, Amsterdam, Netherlands; ^3^Department of Radiology and Nuclear Medicine, Amsterdam Gastroenterology, Endocrinology and Metabolism Research Institute, Amsterdam UMC, University of Amsterdam, Amsterdam, Netherlands; ^4^Clinical Research, Physicians Committee for Responsible Medicine, Washington, DC, United States; ^5^Industry Partner Bond University, Gold Coast, QLD, Australia

**Keywords:** non-communicable disease (NCD), saturated fat, US dietary trends, cardiovascular disease, dietary fat

## Introduction

The article “United States Dietary Trends Since 1800: Lack of Association Between Saturated Fatty Acid Consumption and Non-communicable Diseases,” written by Lee et al. and published in January 2022 in Frontiers in Nutrition (1) has examined the association between dietary saturated fatty acids (SFAs) and non-communicable diseases (NCDs), which is a topic of utmost public health importance. This article claims that there is a lack of association between SFA-consumption and NCDs. This conclusion is not supported by the presented data, it contradicts with robust studies looking at the relationship between dietary fats and disease (2) and with the advice to limit SFA-consumption from the American Heart Association (3).

## Data Collection, Data Quality, Data Processing, and “Food Losses”

First, the article is categorized as a systematic review, while the method section or appendix lacks a systematic search for literature data, as well as inclusion and exclusion criteria for data selection for both literature and database data. Instead, this work should be classified as a longitudinal ecological study, aiming to assess time-related trends in the aggregated prevalence of NCDs and identify population-level dietary factors that might explain these changes over time. A major limitation is that aggregated data cannot be attributed to the individual level. Moreover, using food availability data has the crucial disadvantage as it cannot accurately portray consumption nor distribution of foods within the population (for example among different socioeconomic, age and gender groups). Without replication of the results at the individual level, the association stated by the authors can only be considered hypothesis forming (4) and not as a conclusion extracted from the research presented in the article.

Furthermore, handling of missing data and data processing was not described in the article. For example, it is unclear how the increases in poultry and cheese availability of 550 and 908%, respectively, ultimately arrive at net increases of 136 and 209%. What are the food losses based on? In any case, these losses are not in line with the estimated total global food waste of around 30% (5). In addition, the authors describe that before 1909, no systematic data were available, and there was very little quantitative data on red meat, poultry, dairy, and eggs. These discrepancies are especially critical as meat, poultry, dairy, cheese, and eggs are important sources of SFAs. This adds to the questionable nature of the alleged non-existent relationship between SFAs and NCDs as suggested by the authors. Furthermore, it is unclear which criteria have been used to compile the different product groups in this study. For example, it would have been insightful to distinguish processed from unprocessed grain products. Also, the composition of the group “meat, eggs, and nuts” is not consistent with the primary interest in SFAs. In addition to the unclear handling of data, we also discovered some errors. For example, butter contains 80–85 g of total fat per 100 g (depending on the database consulted). We assume a typing error when stating that butter contains 848 g of total fat per 100 g. Yet, confusingly, Figure 7 suggests that butter contains only ~65 g of fat per 100 g. Also, animal fat, SFAs from all products and added fats/oils are used interchangeably in the article, which causes further confusion for readers. Finally, no attempt to account for confounding variables has been made, such as use of statins, physical activity, smoking, and many others that have changed over the centuries.

## How Much Saturated Fats and How Many Non-Communicable Diseases?

Lastly, the title concludes there is a lack of association between the consumption of SFAs and NCDs. However, no attempt has been made to quantify the SFAs coming from different foods. In fact, Table 2 shows an increase in the consumption of poultry by 413%, dairy products by 15%, and eggs by 241%. The results section also shows an increase in fish and shellfish consumption by 186%, and an increase in intake of processed meat by 3%. All these foods are significant sources of SFAs. Furthermore, the authors do not report any data on NCDs nor perform statistical tests to assess the association between SFAs and NCDs. Interestingly, while the article of Lee et al. suggests an inverse correlation between SFAs and NCDs, Figure 2 shows a relatively stable 18% increase in SFA-consumption while NCDs have 'risen dramatically' according to the authors. Although the authors claim this rise is due to an increase in added plant- and not animal-based fats, given the lack of data on NCDs, quantification of SFAs, and an adequate statistical analysis to support their claim, this conclusion should be interpreted with the utmost caution because it could be detrimental to health.

## Discussion

The title of the article is misleading because the stated conclusion cannot be drawn from the research described in the article. In addition, studies show mortality rates due to cardiovascular disease have declined significantly in recent decades (6) which can be attributed to better treatment, but also to lower intakes of SFAs (7, 8). In fact, a Cochrane systematic review based on randomized controlled trials with human subjects suggested that replacement of SFAs for polyunsaturated fatty acids results in a 27% reduction in cardiovascular events (2).

The aim and hypothesis of the study by Lee et al. were not stated clearly and most importantly, no statistical analysis is described that would show a lack of association. We are concerned that this utterly unsubstantiated title may find its way into the practices of medical doctors and dietitians, who—without proper evidence—may recommend people to eat more saturated fats, and thus damage their health. We therefore ask the authors of the discussed article (1) to provide a thorough analysis between carefully quantified dietary SFAs and NCDs and to account for confounding variables before drawing any conclusions.

## Author Contributions

WW and CJ: conceptualization. WW, CJ, AK, MP, CW, FT, HK, SH, and DS: original draft preparation. DS: supervision. All authors have read and agreed to the published version of the manuscript.

## Conflict of Interest

The authors declare that the research was conducted in the absence of any commercial or financial relationships that could be construed as a potential conflict of interest.

## Publisher's Note

All claims expressed in this article are solely those of the authors and do not necessarily represent those of their affiliated organizations, or those of the publisher, the editors and the reviewers. Any product that may be evaluated in this article, or claim that may be made by its manufacturer, is not guaranteed or endorsed by the publisher.
